# Charge Transfer in Spatially Defined Organic Radical Polymers

**DOI:** 10.1021/acs.chemmater.3c02148

**Published:** 2023-10-30

**Authors:** Ting Ma, Evan Fox, Miao Qi, Cheng-Han Li, K. A. Niradha Sachithani, Khirabdhi Mohanty, Daniel P. Tabor, Emily B. Pentzer, Jodie L. Lutkenhaus

**Affiliations:** † Artie McFerrin Department of Chemical Engineering, Texas A&M University, College Station, Texas 77843, United States; ‡ Department of Chemistry, Texas A&M University, College Station, Texas 77843, United States; § Department of Materials Science and Engineering, Texas A&M University, College Station, Texas 77843, United States

## Abstract

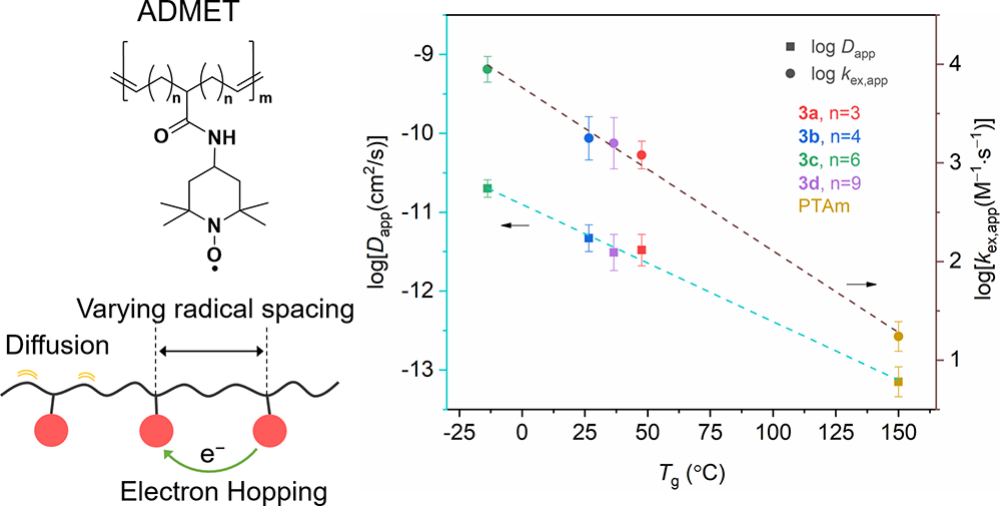

Charge transfer in nonconjugated redox-active polymers is influenced by redox site proximity and polymer flexibility, but it is challenging to observe these effects independently. In this work, spatially defined radical-containing polymers are synthesized by using acyclic diene metathesis (ADMET) polymerization of α,ω-dienes bearing a central activated ester. Postpolymerization functionalization with 4-amino-2,2,6,6-tetramethylpiperidine-1-oxyl (4-amino-TEMPO) introduces TEMPO radical groups onto the polymer backbone through amide linkages to yield spatially defined polymers with radical units every 9, 11, 15, and 21 carbons. Increased radical spacing leads to reduced spin–spin coupling and increased chain flexibility. The glass transition temperatures (*T*_g_) range from 47.6 to −13.8 °C, depending on the radical spacing. The spatially defined TEMPO-substituted polymer with a spacing length of 15 carbons displays the lowest *T*_g_ and the shortest hopping distance, as shown through molecular dynamics simulations. Also, this polymer displays kinetics 1000 times faster than the commonly studied TEMPO-containing polymer poly(2,2,6,6-tetramethylpiperidinyloxy-4-ylacrylamide) (PTAm). Remarkably, comparison of the diffusion and kinetics attributed to the redox reaction reveals that both the apparent diffusion coefficient and the self-exchange reaction rate constant are correlated to the polymer’s *T*_g_ as log[*D*_app_] and log[*k*_ex,app_] ∼ *T*_g_, respectively. Critically, these data demonstrate that controlling the spacing of redox-active groups along a polymer backbone strongly influences backbone flexibility and radical packing, which leads to synergetic improvements in the charge transfer kinetics of nonconjugated redox-active polymers.

## Introduction

Nonconjugated redox-active polymers (NC-RAPs) are promising electrode materials for organic batteries, which are important for advancing sustainable and circular energy storage systems.^1−6^ The electron transfer process in NC-RAPs can be explained by a Marcus–Hush-type electron-hopping mechanism with Brownian motion of the redox-active sites, as described by a diffusion-cooperative model.^7−9^ The former process is influenced by the site-to-site distance, and the latter is influenced by the polymer backbone’s flexibility. It is challenging to decouple the contributions of both processes because adjusting one factor through synthesis also adjusts the other. Therefore, only a few reports address relationships among backbone identity, physical properties, and structure.^9−11^ Further, there is an incomplete understanding of how redox site spacing and polymer flexibility influence each other and, as a result, the charge transfer kinetics.

The nitroxide radical is commonly studied as an active unit in organic battery electrodes because of its shelf-stability, air and moisture tolerance, battery-like redox chemistry, and the ease of using it as a molecular building block.^3,4,12−15^ 2,2,6,6-Tetramethylpiperidine 1-oxyl (TEMPO) and a few of its modified derivatives are commercially available, making them relatively easy to covalently integrate into macromolecules using reactions that leave the nitroxide unaltered.^16,17^ TEMPO-substituted nonconjugated polymers have good chemical and electrochemical stabilities owing to the highly localized unpaired electron and the steric hindrance of the TEMPO’s four methyl groups.^5,18−20^ As a representative example, poly(2,2,6,6-tetramethylpiperidinyloxy-4-ylacrylamide (PTAm) is usually synthesized by controlled radical polymerization (radical content 70–80%)^21^ and exhibits high redox potentials (∼3.6 V vs Li/Li^+^), reversible charge storage capacity (>100 mA h/g for 1000+ cycles), and rapid electron transfer kinetics (electron transfer rate constant of 10^–1^ cm s^–1^) with radical concentrations higher than 1 mol/L.^6,9,22^ The importance of chain flexibility in the solid-state conductivity of NC-RAPs was highlighted by Joo et al.,^23,24^ who reported that TEMPO-substituted polymers with a subambient *T*_g_ had an electrical conductivity of 28 S/m after thermal annealing. However, the importance of chain flexibility as it relates to *T*_g_ has not yet been fully proven or examined in the context of energy storage in which the polymer undergoes a redox reaction.

Here, we synthesized a series of spatially defined TEMPO-containing polymers by using acyclic diene metathesis (ADMET) polymerization for the first time.^25,26^ We prepared polymers with radical units every 9, 11, 15, and 21 carbons along the backbone using different α,ω-dienyl monomer lengths (*n* = 3, 4, 6, and 9, respectively). Spin–spin coupling was examined by using electron paramagnetic resonance (EPR) spectroscopy and compared to the mean hopping distance and degree of percolation, as revealed by molecular dynamics (MD) simulations. The charge transfer kinetics, measured using electroanalytical chemistry, are discussed in the context of the polymers’ *T*_g_ values and radical spacing, in which it is shown that the polymer with the lowest *T*_g_ (*n* = 6) has the shortest hopping distance and the fastest charge transfer kinetics. The results are discussed in the context of scaling electron transfer phenomena with the diffusion behavior of the polymer.

## Results and Discussion

**Figure 1:**
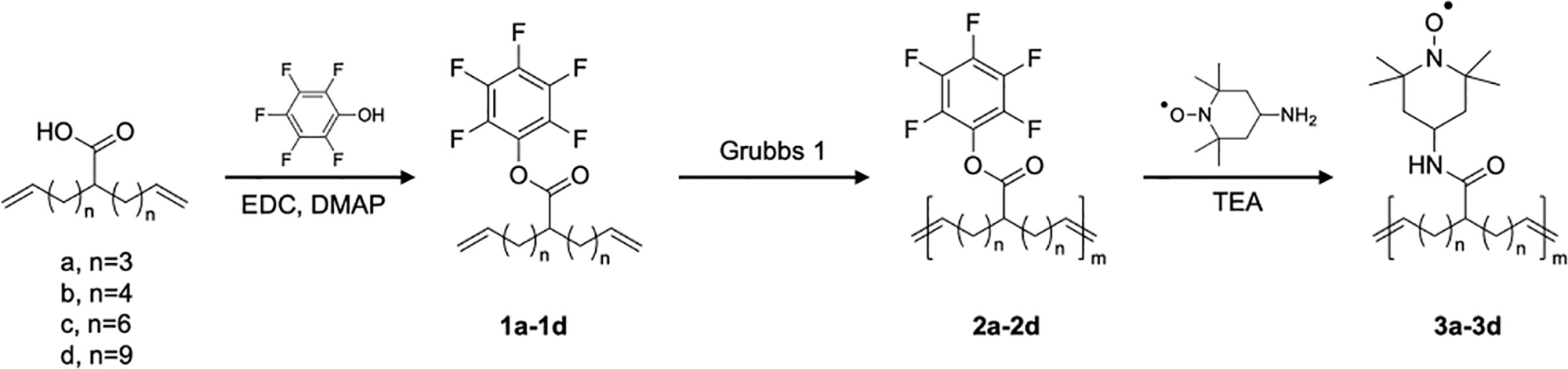
Synthetic approach for the preparation of the spatially defined radical-containing polymers using ADMET polymerization and postpolymerization modification.

A series of polymers with different numbers of backbone carbons between neighboring TEMPO groups was synthesized by ADMET polymerization; see Figure 1 and Supporting Information for details. α,ω-Dienes of different lengths bearing pendant carboxylic acids on the central carbon were synthesized from a literature-reported procedure,^27^ and the carboxylic acid groups were transformed to activated esters (pentafluorophenyl ester) via 1-ethyl-3-(3-(dimethylamino)propyl)carbodiimide (EDC) coupling. ADMET polymerizations of monomers **1a**–**1d** proceeded with limited catalyst decomposition when performed under reduced pressure to facilitate the removal of the ethylene byproduct and ensure conversion to higher molar mass, with polymers of similar molecular weight prepared (∼10 kg/mol). ^13^C NMR (nuclear magnetic resonance) spectroscopy of polymers **2a**–**2d** revealed that the *trans*/*cis* ratio of the polymer backbone alkene groups was ∼2:1, which is consistent with literature-reported values for ADMET polymerizations with Grubbs first generation metathesis catalyst (Figures S1–S6). Postpolymerization, the activated ester was reacted with 4-amino-TEMPO to produce the radical-containing polymers **3a**–**3d** with radical spacing defined by *n* = 3, 4, 6, and 9 (TEMPO units every 9, 11, 15, and 21 carbons). Using **2a** and **3a** as a representative example (Figure S7), this chemical transformation was monitored using Fourier transform infrared (FTIR) spectroscopy by the disappearance of the aromatic C–C bond signal at 1517 cm^–1^ and ester C=O signal at 1782 cm^–1^ and the appearance of an amide C=O signal at 1645 cm^–1^. Further, the FTIR spectra for the targeted polymer contained an amide N–H bond at 3286 cm^–1^. The FTIR spectra before and after the reaction indicate that the substitution reaction went to full conversion (e.g., complete consumption of the activated ester and formation of the amide, with no hydrolysis product).

The radical content and the interaction between the unpaired electron spins of neighboring TEMPO moieties of polymers **3a**–**3d** were examined by using EPR spectroscopy with a concentration of 1 mM in chloroform. The EPR spectra were double-integrated, since an EPR spectrum is a first derivative, and compared with 4-amino-TEMPO (1 mM solution in chloroform) to calculate radical content. All polymers had a radical content of >95%, which is higher than past-reported radical polymers synthesized by postpolymerization oxidation reactions of the tetramethylpiperidine amine, as the synthesis here does not require oxidation of N–H to N-oxyl radicals.^21^

**Figure 2:**
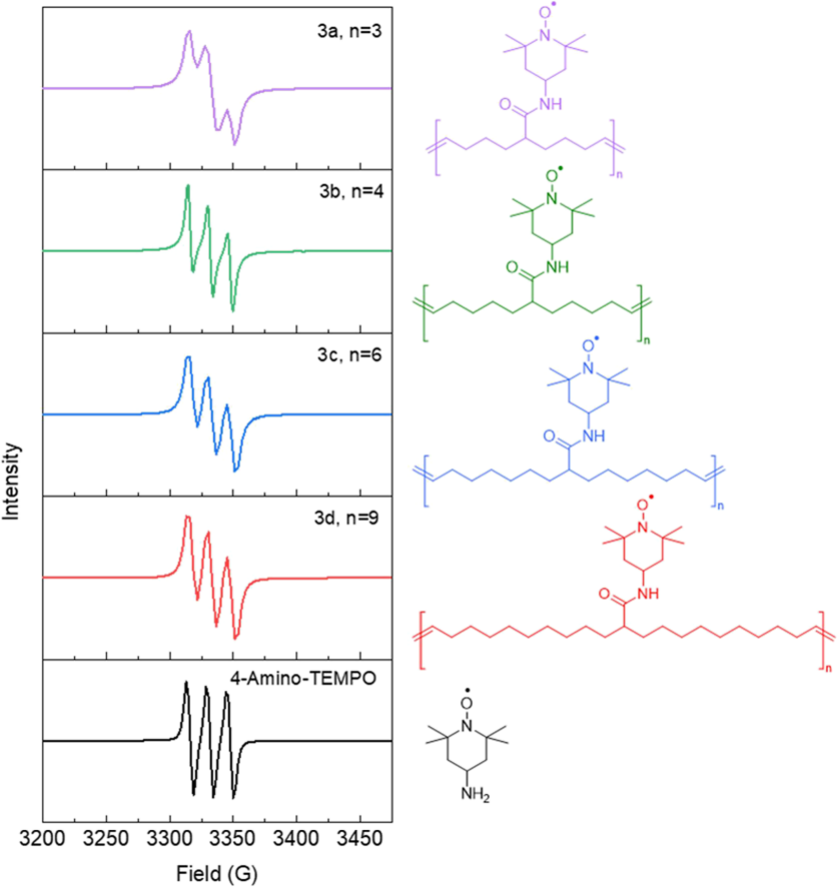
Solution EPR spectra and molecular structure of each polymer and 4-amino TEMPO (1 mM in chloroform).

As shown in Figure 2, the unpaired electron of 4-amino-TEMPO manifests as a triplet multiplicity in solution, which signifies its interaction with nitrogen nuclei possessing a spin quantum number of *I* = +1. This interaction accounts for the observed splitting pattern, which follows the formula (2*I* + 1). Comparing the EPR splitting patterns of the synthesized polymers with varying radical spacing, a similar triplet multiplicity is observed in the spectra of **3b**–**3d** (*n* = 4, 6, and 9), whereas **3a** (*n* = 3) exhibits a broad doublet multiplicity. This result can be ascribed to the shorter spacing length between the unpaired electrons in **3a**, which leads to heightened spin–spin interaction, resulting in line broadening effects. Consequently, the ability to discern hyperfine splitting decreases, giving rise to the observed doublet pattern. In highly concentrated solutions, the interaction between unpaired electrons becomes substantial, rendering the resolution of hyperfine splitting impossible.^28^ As a consequence, the observed signal can be reduced to a singlet multiplicity.^29,30^

To investigate the effect of radical spacing on the thermal properties of the polymers, differential scanning calorimetry (DSC) was performed (Figure S8). Table 1 lists the *T*_g_ for polymers **3a**–**3d** and PTAm, which is a commonly used NC-RAP with TEMPO groups on every other carbon of the backbone. Upon increasing the radical spacing from *n* = 3 to *n* = 6, the *T*_g_ decreased from 47.6 to −13.8 °C. However, further increasing the spacing to *n* = 9 led to an increase of the *T*_g_ to 36.9 °C. In comparison, PTAm has fewer backbone carbons between radical groups and thus a higher radical density (*C*_E_), leading to a much higher *T*_g_ (∼150 °C). These results indicate that increasing the number of carbons in the backbone from *n* = 3 to *n* = 6 led to decreased intermolecular interactions, which lowers the barriers for chain motion, thus lowering the *T*_g_. When increasing the radical spacing to *n* = 9, the *T*_g_ increased due to the longer hydrocarbon segments, which can locally order with one another. Previous work^31,32^ found similar results for spatially defined polyethylene-based ionomers in which *T*_g_ shows an apparent dependence on both spacer length and crystallinity, which appeared at saturated analogues with 21 carbons (*n* = 9). Taken together, *T*_g_ is dependent on both the spacing length and the radical density of the polymer.

**Table 1:** Comparison of Polymer Properties with Varying Radical Spacing

polymer	*T*_g_ (°C)	*C*_E_ (mol/cm^3^)	mean δ (Å)	*D*_app_ (cm^2^/s)	*k*_ex,app_ (M^–1^ s^–1^)
**3a**, *n* = 3	47.6	2.86 × 10^–3^	7.64^a^	3.33 × 10^–12^	1.20 × 10^3^
**3b**, *n* = 4	26.6	2.65 × 10^–3^	7.77^a^	4.69 × 10^–12^	1.76 × 10^3^
**3c**, *n* = 6	–13.8	2.31 × 10^–3^	7.62^a^	1.99 × 10^–11^	8.90 × 10^3^
**3d**, *n* = 9	36.9	1.93 × 10^–3^	7.80^a^	3.11 × 10^–12^	1.59 × 10^3^
**PTAm**	∼150^33^	3.92 × 10^–3^	7.93^34^	7.14 × 10^–14^	1.74 × 10^1^

a From MD simulations.

**Figure 3:**
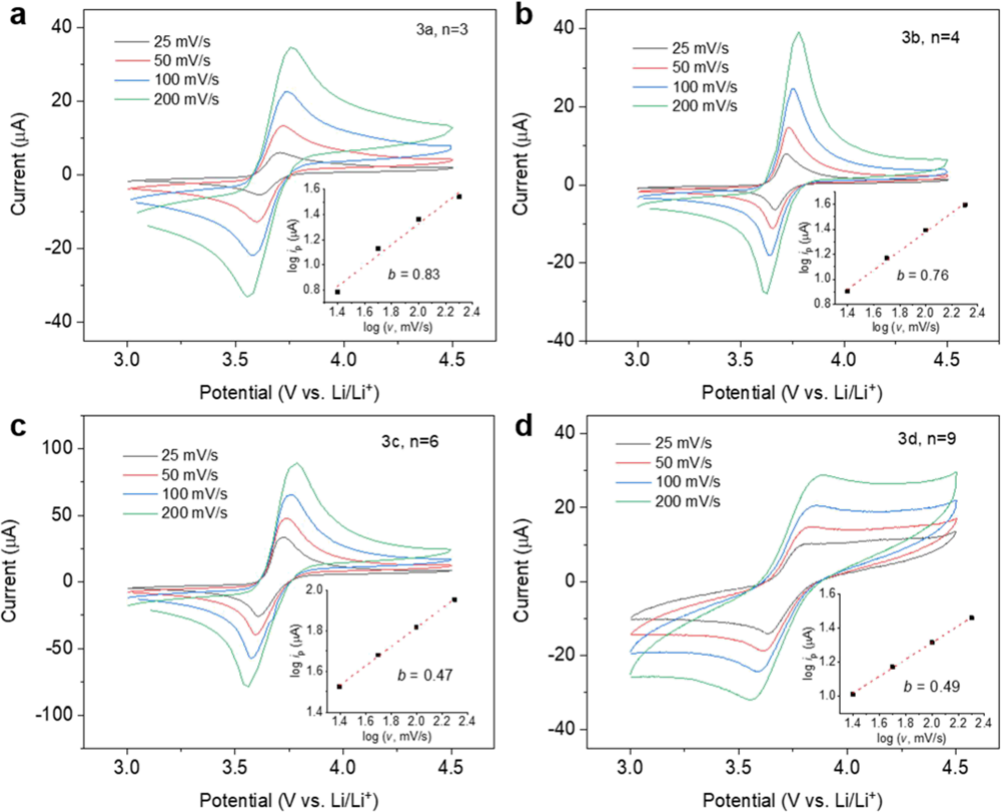
Cyclic voltammetry of the TEMPO-containing polymers with varying radical spacings (a) **3a**, *n* = 3, (b) **3b**, *n* = 4, (c) **3c**, *n* = 6, and (d) **3d**, *n* = 9. The working electrode was prepared by drop-casting the polymer onto a glassy carbon electrode (areal mass loading was ∼0.1 mg/cm^2^). The electrolyte was 0.5 M lithium triflate (LiOTf) in propylene carbonate (PC). Li metal strips were used as the counter and reference electrodes. Insets show the log–log plot of the peak current versus the scan rate to obtain the *b*-value of each polymer.

To understand the effect of radical spacing on charge transport, we investigated the redox kinetics of these polymers as films by performing cyclic voltammetry (CV) at different scan rates from 25 to 200 mV s^–1^ (Figure 3). These polymers all show one pair of reversible, symmetric redox couples occurring around the same redox potential, correspond to anion transfer associated with the TEMPO radical/oxoammonium cation redox couple (*E*_1/2_ ≈ 3.7 V vs Li/Li^+^). The current density was stable during CV cycling, indicating no visible dissolution and that the polymer layers stayed at the electrode surface (Figure S9). The peak separation, Δ*E*_p_, increased with the radical spacing of *n* = 3 (93 mV), *n* = 4 (102 mV), *n* = 6 (118 mV), and *n* = 9 (177 mV), mainly owing to the decreased radical density.

To understand the nature of the electrochemical reaction, the CV responses were analyzed according to the power law: *i*_p_ = *av*^*b*^, where *a* is an alterable parameter and the *b*-value describes the reaction–diffusion behavior. Generally, a *b*-value of 0.5 suggests a diffusion-controlled electrochemical process, and a value of 1.0 indicates a surface-controlled, non-Faradaic electrochemical process.^12,35,36^ As shown in the insets of Figure 3a–d, the *b*-values of the polymers are 0.83, 0.76, 0.47, and 0.49 for polymers **3a**, **3b**, **3c**, and **3d**, respectively. The decrease in the *b*-value with increasing radical spacing indicates that the redox-process changes from a mixed surface and diffusion-controlled process to a fully diffusion-controlled process. This result is consistent with the spin–spin coupling responses shown in Figure 2. Specifically, polymers **3a** displayed doublets in the EPR responses, whereas polymers **3b**–**3d** displayed triplets, suggesting that the reaction–diffusion behavior can be influenced by the strength of radical–radical coupling.

**Figure 4:**
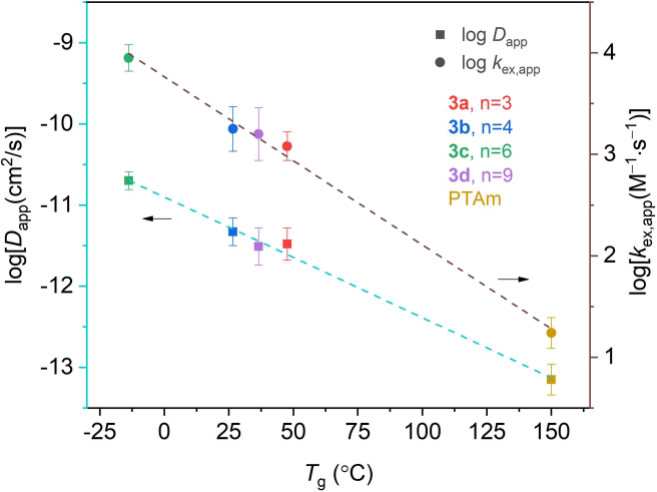
Comparison of log[*D*_app_ (cm^2^ s^–1^)] and log[*k*_ex,app_ (M^–1^ s^–1^)] with *T*_g_ for polymers of different radical spacing. The data points are the mean value estimated from three measurements, and the error bar is the standard deviation. The lines represent linear fits (log[*D*_app_] = −0.015*T*_g_ – 10.9, *R*^2^ = 0.996; log[*k*_ex,app_] = −0.017*T*_g_ + 3.76, *R*^2^ = 0.996).

To further evaluate the redox process, the apparent diffusion coefficient (*D*_app_) and self-exchange reaction rate constant (*k*_ex,app_) were quantified using the Randles–Sevcik and Dahms–Ruff equations and using hopping distances from simulations described below and in the Supporting Information. Figure 4 shows the relationship between *D*_app_ and *k*_ex,app_ with *T*_g_ for the spatially defined polymers and PTAm. Polymer **3c** exhibited the fastest kinetics—even 1000× higher than that of PTAm (Figure S10), even though **3c**’s radical density is significantly lower than polymers **3a, 3b,** and PTAm). Interestingly, we observed a linear relationship with log[*D*_app_] and log[*k*_ex,app_] with *T*_g_, which was not reported elsewhere. In considering the meaning of this, we note that each system was measured at room temperature (*T* = 25 °C), such that diffusion scales as log[*D*_app_] ∼ *T*_g_/*T* in an Arrhenius-type relationship. In the case of diffusion-limited redox reactions, *k*_ex,app_ is proportional to the physical diffusion of the redox-active species, *k*_ex,app_ ∼ *D*_phys_.^3^ Because we observe that log[*D*_app_] ∼ *T*_g_, we conclude that *D*_app_ is proportional to *D*_phys_ for this system; see the Supporting Information for extended explanation. We note that the polymer is expected to swell with the electrolyte, leading to a depression of the *T*_g_ (i.e., plasticization); therefore, the observed electrochemistry is likely that of a polymer in its rubbery, plasticized state, with the true *T*_g_ of the polymer in situ uncertain. Taken together, these results show that an increased polymer chain mobility (by decreasing the *T*_g_) promotes electron exchange through the Brownian motion of radical sites.

**Figure 5:**
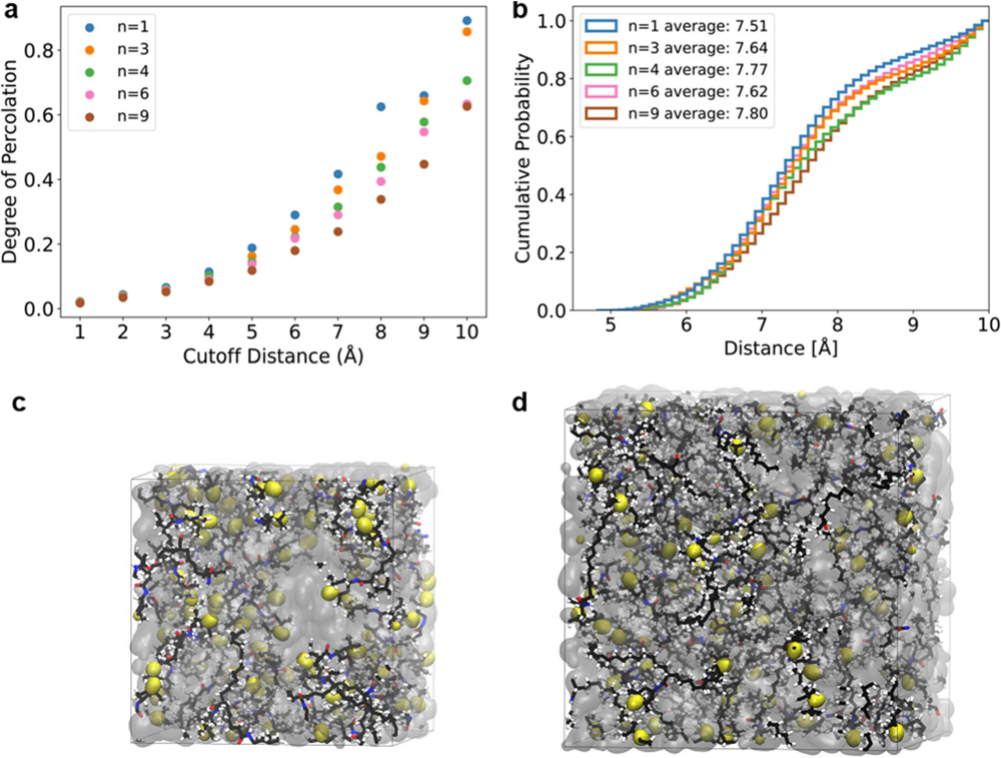
(a) Calculated degree of percolation of each polymer as a function of the nearest-neighbor cutoff distance. (b) Cumulative distribution of the nearest-neighbor hopping distances from MD simulations for each polymer. MD snapshots for (c) *n* = 1 and (d) *n* = 9 with N–O· atoms on TEMPO units shown as yellow surfaces, solvent molecules as translucent gray, and all other polymer bonds and atoms explicitly shown.

To better understand the effect of TEMPO spacing on kinetics, MD simulations were conducted to calculate the cumulative distribution of hopping distance and degree of percolation for polymers with *n* = 1, 3, 4, 6, and 9 (the latter four correspond to polymers **3a–3d**, respectively), as shown in Figure 5a,b and Supporting Information. Because electron hopping between TEMPO units is considered negligible over 10 Å,^9,37^ we focused on pairs of TEMPO units that were within 10 Å of each other and calculated the mean hopping distance (δ), along with the count of TEMPO pair interactions (within a given distance, Table S1). We followed the procedure from Joo et al.^23^ to calculate the degree of percolation with a lattice spacing up to 10 Å. As shown in Figure 5a, the degree of percolation decreases from 1 to 9, consistent with the observation of the decreasing number of TEMPO pairs (Table S1). The contrast in percolation between the different spacings is more pronounced at cutoff distances over 6 Å. Inspection of MD simulation snapshots for polymers with *n* = 1 and *n* = 9 clearly reveals that the TEMPO units are closer to each other in the case of *n* = 1 (Figure 5c,d).

For polymers **3a–3d**, the TEMPO pair counts and degree of percolation decreased with increasing radical spacing. Although the order does not exactly follow the trend of their charge transfer kinetics, the trend of charge kinetics does follow the order of the cumulative distribution of hopping distances over 6.6 Å in Figure 5b. A higher probability of occurrence in shorter distances for polymer **3a** is attributed to its shorter radical spacing. Polymer **3c**, with moderate TEMPO pair counts and degree of percolation, shows a much faster charge transfer process than the others. In Marcus–Hush theory, the electron transfer rate is related to the hopping distance. We were thus surprised that **3c** exhibited the shortest average hopping distance from the MD simulation, which correlates with **3c** having the fastest kinetics (Figure S11). We ascribed **3c**’s shorter average hopping distance to its lower *T*_g_ relative to those of the other polymers. Therefore, this result indicates that charge transfer in this series of polymers is influenced by the physical diffusion of the redox sites, affecting the TEMPO pair distance and the probability of their pairwise interaction.

## Conclusions

In conclusion, a series of spatially defined TEMPO-substituted radical polymers were synthesized by using ADMET polymerization for the first time. The obtained polymers **3a–3d** have backbone spacing lengths of 9, 11, 15, and 21 carbon (*n* = 3, 4, 6, and 9). The increase in radical spacing reduced spin–spin interactions and decreased intermolecular interactions and radical density in the polymer, and the splitting patterns changed from doublet multiplicity polymer (**3a**) to triplet multiplicity polymers (**3b–3d**). The spatially defined polymers exhibited varying *T*_g_’s, ranging from 47.6 to −13.8 °C, with an apparent dependence on both spacing length and the radical density in the polymer. Polymer **3c** (*n* = 6), which has the lowest *T*_g_ of −13.8 °C and the shortest hopping distance of 7.62 Å (from MD simulations), exhibited roughly 10× and 1000× enhancement in kinetics relative to the other polymers and to PTAm, respectively. A linear relationship of log[*D*_app_] and log[*k*_ex,app_] with *T*_g_ was revealed, which highlights the importance of polymer mobility in the redox process.

These findings indicate that lowering the *T*_g_ of the redox-active polymer has the advantage of promoting the kinetics of electron exchange, which will have a direct impact on the advancement of fast-charging batteries. We showed that decreasing the density of the redox-active sites resulted in a lower *T*_g_ up to the point, but by doing so, the theoretical capacity of the polymer (related to *C*_E_) decreased as well. Therefore, future work should focus on lowering the *T*_g_ while maintaining a high theoretical capacity. This study provides both a synthesis pathway for spatially defined radical polymers and a design principle to improve the electrochemical properties of NC-RAPs.

## Experimental Section

### Materials

All chemicals were used as received from Sigma-Aldrich unless otherwise noted.

### Synthesis and Characterization

The spatially defined TEMPO-containing polymers were synthesized using ADMET polymerization. Detailed procedures can be found in Supporting Information. FTIR spectroscopy was performed using a JASCO FTIR spectrometer, model FTIR-4600LE MidIR. ^1^H, ^13^C, and ^19^F NMR spectra were recorded on a Bruker 400 spectrometer. EPR spectroscopy experiments were completed using a Bruker EMX-EPR spectrometer. Glass transition temperatures were measured by modulated differential scanning calorimetry on a Q200 DSC (TA Instruments) with a heat–cool–heat cycle. The glass transition temperature was taken as the inflection point of the second heating cycle. Molecular weight was obtained by gel permeation chromatography (TOSOH high-temperature GPC with either trichlorobenzene or tetrahyrofuran).

### Electrochemical Analysis

Electrochemical measurements were conducted using a three-electrode cell in an argon-filled glovebox at room temperature. Separate Li metal strips were used as reference and counter electrodes, respectively. A polymer-coated glassy carbon was used as the working electrode for CV in a 0.5 M LiOTf/PC electrolyte (5 mL). The working electrode was prepared by drop-casting polymer/CHCl_3_ solution (1 mg/mL, 20 μL) onto the glassy carbon surface, followed by vacuum drying. The typical areal mass loading was around 0.1 mg/cm^2^. A Gamry Interface 1000 instrument was employed for electrochemical measurements. The apparent diffusion coefficient, *D*_app_, was calculated using the Randles–Sevcik equation. Based on the obtained *D*_app_, the apparent self-exchange rate constant, *k*_ex,app_, was calculated using the Dahms–Ruff equation. Kinetic analysis can be found in the Supporting Information.

## Supporting Information Available

Synthesis procedures and characterizations; kinetic analysis; MD simulation computational details; peak and mean values of hopping distances within 10 Å and the number of TEMPO dimers; ^1^H NMR spectroscopy of **1b**; ^13^C NMR spectroscopy of **1b**; ^19^F NMR spectroscopy of **1b**; ^1^H NMR spectroscopy of **2b**; ^13^C NMR spectroscopy of **2b**; ^19^F NMR spectroscopy of **2b**; FTIR spectra of **2a** and **3a**; DSC thermograms of each polymer; CV of the TEMPO-containing polymers; CV of PTAm; and comparison of log[*D*_app_] and log[*k*_ex,app_] with average hopping distance δ for polymers of different radical spacing (PDF)
