# Supplementary material for: Charge Transfer in Spatially Defined Organic Radical Polymers
Source: Chem Mater. 2023 Oct 30;35(21):9346–51. doi: 10.1021/acs.chemmater.3c02148 (PMC10862473; doi:10.1021/acs.chemmater.3c02148)
Supplement: Supplementary file 1 — cm3c02148_si_001.pdf [file cm3c02148_si_001.pdf]

Supplementary Information

Charge Transfer in Spatially Defined Organic Radical Polymers

Ting Ma[1,+], Evan Fox[2,+], Miao Qi[1,2], Cheng-Han Li[2], K. A. Niradha Sachithani[2], Khirabdhi Mohanty[1], Daniel P. Tabor[2,*], Emily B. Pentzer[2,3*], and Jodie L. Lutkenhaus[1,3,*]

[1]Artie McFerrin Department of Chemical Engineering, Texas A&M University, College Station, TX 77843, USA

[2]Department of Chemistry, Texas A&M University, College Station, TX 77843, USA

[3]Department of Materials Science and Engineering, Texas A&M University, College Station, TX 77843, USA

[+]These authors contributed equally

*Corresponding author: daniel_tabor@tamu.edu; emilypentzer@tamu.edu; jodie.lutkenhaus@tamu.edu

Synthesis Procedures

Synthesis of 1a. A representative general procedure is described. Starting material 2-(pent-4-en-1-yl)hept-6-noic acid was synthesized from literature-reported procedures. To a 2-neck 250 mL round bottom flask, 2-(pent-4-en-1-yl)-hept-6-enoic acid (0.981 g, 5.00 mmol) pentalfluorophenol (2.30 g, 12.5 mmol) and 4-dimethylaminopyridine (DMAP) (0.611g, 5.0 mmol) were dissolved in dry dichloromethane (30 mL). The flask was then put under nitrogen and cooled to 0 °C. To the side neck, a suspension of EDC•HCl (2.40 g, 12.5mmol) in dry dichloromethane (20 mL) was added dropwise. After the addition, the reaction mixture was allowed to warm up to room temperature and was stirred for an additional 24 hours. The reaction was monitored with TLC in 10/90 ethyl acetate/hexanes. After the reaction was complete, the solvent was removed under reduced pressure using a rotavap. The product was further purified with column chromatography with silica and 10/90 ethyl acetate/hexanes to afford a colorless oil. Yield: 87%. ^{1}H-NMR (400 MHz, $CDCl_3$) δ 5.79 (*m*, 4*H*), 4.97 (*m*, 2*H*), 2.70 (*m*, 1*H*), 2.09 (*q*, 4*H*), 1.58-1.83 (*m*, 4*H*) 1.49 (*m*, 4*H*) ppm. ^{13}C-NMR (100 MHz, $CDCl_3$) δ 172.8, 142.4, 139.8, 136.6, 133.9, 118.08, 44.9, 35.6 ppm. ACPI-MS, $[M+H]^+$ *m/z* calculated: 363.1378 found: 363.1372

Synthesis of 1b. 1b was synthesized following a procedure similar to that for **1a,** instead using 2-(hex-5-en-1-yl)oct-7-enoic acid as the starting material**.** Yield: 74%. ^{1}H-NMR (400MHz, $CDCL_3$) δ 5.8 (*m*, 2H), 4.98 (*m*, 4H), 2.70 (*m*, 1H), 2.07 (*q*, 4H), 1.62-1.77 (*m*, 4H), 1.43 (*m*, 8H) ppm. ^{13}C-NMR (100 MHz, $CDCl_3$) δ 172.4, 141.4, 139.5, 138.7, 138.1, 125.3, 114.7, 45.6, 33.6, 32.4, 28.8, 26.8 ppm. ACPI-MS, $[M+H]^+$ *m/z* calculated:391.1691 found:391.1686

Synthesis of 1c. 1c was synthesized following a procedure similar to that described for **1a,** instead using 2(oct-7-en-1-yl)dec-9-enoic acid as the starting material**.** Yield: 81%. ^{1}H-NMR (400 MHz, $CDCl_3$) δ 5.83 (*m*, 4*H*), 4.96 (*m*, 2*H*), 2.72 (*m*, 1*H*), 2.41 (*q*, 4*H*), 1.53-1.78 (*m*, 4*H*), 1.27-1.81 (*m*,

16*H*) ppm. ^{13}C-NMR (100 MHz, $CDCl_3$) δ 172.8, 142.2, 139.4, 135.6, 134.0, 119.1, 114.2, 46.1, 34.2, 32.9, 29.9, 29.3 27.7 ppm. ESI-MS, $[M+H]^+$ *m/z* calculated: 447.2317 found: 447.216

Synthesis of 1d. 1d was synthesized following a procedure similar to that described for **1a,** instead using 2-(undec-10-en-1-yl)tridec-12-enoic acid as the starting material. Yield: 76%. ^{1}H-NMR (400 MHz, $CDCl_3$) δ 5.81 (*m*, 4*H*), 5. (*m*, 2*H*), 2.70 (*m*, 1*H*), 2.40 (*q*, 4*H*), 1.54-1.78 (*m*, 4*H*), 1.26-1.79 (*m*, 28*H*) ppm. ^{13}C-NMR (100 MHz, $CDCl_3$) δ 172.9, 142.4, 139.8, 135.6, 134.0, 118.08, 114.6, 46.1, 34.2, 32.9, 29.9, 29.3 27.7 ppm. ESI-MS, $[M+H]^+$ *m/z* calculated: 531.3256 found: 531.3250

ADMET polymerization

The monomer was dried by filtering through a dry alumina plug in the glove box. **1a** (1.00 g, 2.76 mmol) was added to a 50 mL Schlenk flask. Grubb's 1st generation catalyst was dissolved in THF (1 ml) and added to the monomer. The flask was then put under reduced pressure on the Schlenk line for 5 min, sealed, and heated to 50 °C with stirring. After 3 days, full monomer conversion was observed by NMR, and the polymer was precipitated into cold methanol to afford a light brown solid. This general procedure was used for **2a-2d**; with the exception being that **2b** polymerization was run under constant flowing nitrogen instead of a vacuum.

Synthesis of **2a**. Yield: 76%. ^{1}H-NMR (400 MHz, $CDCl_3$) δ 5.81 (*m*, 4*H*), 5. (*m*, 2*H*), 2.70 (*m*, 1*H*), 2.07 (*m*, 4*H*), 1.54-1.78 (*m*, 4*H*), 1.36-1.54 (*m*, 4*H*) ppm. ^{13}C-NMR (100 MHz, $CDCl_3$) δ 172.3, 142.6, 139.8, 138.2, 136.7, 134.0, 130.3, 129.7 125.3, 115.1, 45.5, 33.6, 32.4, 32.0, 27.2, 26.5 ppm.

Synthesis of **2b**. Yield:74%. ^{1}H-NMR (400MHz, $CDCl_3$) δ 5.81 (m), 5.38 (*t*, 2H) 5. (*m*), 2.68 (*m*, 1H) 2.01 (*m*, 4H), 1.54-1.78 (*m,* 4H). 1.31-1.46 (*m*, 8H) ppm. ^{13}C-NMR (100 MHz, $CDCl_3$) δ 172.4, 141.4, 139.5, 138.7, 138.1, 130.4, 129.8, 125.3, 45.7, 33.6, 32.5, 2875, 26.9 ppm.

Synthesis of **2c**. Yield: 81%. ^{1}H-NMR (400 MHz, $CDCl_3$) δ 5.81 (*m*, 4*H*), 5. (*m*, 2*H*), 2.70 (*m*, 1*H*), 1.96 (*q*, 4*H*), 1.54-1.78 (*m*, 4*H*), 1.26-1.79 (*m*, 16*H*) ppm. ^{13}C-NMR (100 MHz, $CDCl_3$) δ 172.9, 142.4, 139.8, 135.6, 134.0, 118.08, 114.6, 46.1, 34.2, 32.9, 29.9, 29.3 27.7 ppm.

Synthesis of **2d**. Yield: 76%. ^{1}H-NMR (400 MHz, $CDCl_3$) δ 5.81 (*m*, 4*H*), 5. (*m*, 2*H*), 2.70 (*m*, 1*H*), 1.96 (*q*, 4*H*), 1.54-1.78 (*m*, 4*H*), 1.26-1.79 (*m*, 28*H*) ppm. ^{13}C-NMR (100 MHz, $CDCl_3$) δ 172.9, 142.4, 139.8, 135.6, 134.0, 118.08, 114.6, 46.1, 34.2, 32.9, 29.9, 29.3 27.7 ppm.

Synthesis of 3a. Activated ester polymer **2a** (0.181 g, 0.500 mmol) was dissolved in dry dichloromethane (10 mL) in a 50 mL 2-neck round bottom flask, and the solution was put under nitrogen. 4-amino-TEMPO (0.428 g, 2.5 mmol) and triethyl amine (0.110 mL, 0.8 mmol) were dissolved in dry dichloromethane (10 mL) and added to the reaction flask dropwise. To aid conversion ~3mL of DMSO was added to the reaction mixture. The reaction mixture was allowed to stir at room temperature for 24 hours under nitrogen. The reaction was monitored using FTIR spectroscopy to track the 5F-phenyl ester conversion. The reaction mixture was washed with 10% HCl solution (3 × 25 mL). The organic layer was dried over $MgSO_4$ and condensed under reduced pressure with rotavap and precipitated into ethanol/water solution (1/2 v/v). The product was collected as an orange powder and vacuum dried. Yield: 83%

Synthesis of **3b**. **3b** was synthesized following the procedure described for **3a** but using polymer **2b**. Yield:74%

Synthesis of **3c**. **3c** was synthesized following the procedure described for **3a** but using polymer **2c**. Yield:87%

Synthesis of **3d**. **3d** was synthesized following the procedure described for **3a** but using polymer **2d**. Yield: 67%

Kinetics Analysis

To obtain the apparent diffusion coefficient of electron transfer, D_{app}, the peak current i_p from cyclic voltammetry was plotted against the square root of scan rate, $v^{1/2}$. Then, D_{app} was obtained using the Randles-Sevcik equation[1, 2]:

$$i_p = 0.4463nFAC_E\sqrt{\frac{nFvD_{app}}{RT}} \quad (1)$$

where i_p is the peak current (A), n is the number of electrons transferred in the redox event (n = 1), F is Faraday's constant (96,485 C·mol^{-1}), A is the electrode area (cm^2), C_E is the concentration of the redox species (mol/cm^3), v is the scan rate (V/s), D_{app} is the diffusion coefficient (cm^2/s), R is the universal gas constant (8.314 J·K^{-1}·mol^{-1}) and T is the absolute temperature (K).

The homogeneous electron self-exchange rate constant $k_{ex,app}$ can be calculated from the Dahms-Ruff equation[2, 3]:

$$D_{app} = \tfrac{1}{6}C\delta^2 k_{ex,app} \quad (2)$$

δ is the average hopping distance, which was obtained from MD simulations, listed in **Table 1**.

The apparent self-exchange rate constant has contributions from the activation-limited rate constant (k_{act}) and the diffusion-limited rate constant (k_{diff}):

$$\frac{1}{k_{ex,app}} = \frac{1}{k_{act}} + \frac{1}{k_{diff}} \quad (3)$$

If diffusion of the redox-active species is the rate-limiting species, then activation can be neglected and $k_{ex,app} \approx k_{diff}$. By the diffusion-cooperative model:

$$k_{diff} = 16aD_{phys}N_A \quad (4)$$

By this comparison $k_{ex,app} \sim D_{phys}$. In the present work we observed that $k_{ex,app} \sim D_{app}$, which leads us to the conclusion that diffusion is largely dominated by that of the redox species – in this case, the polymer chain.

Molecular dynamics simulation computational details

Atomistic molecular dynamics (MD) simulations were carried out with LAMMPS (3 Mar 2020 version)[4] software package using OPLS-2005[5] force field extracted by the ffld_server toolkit from the Schrodinger software package's utilities.[6] The force field parameters for the TEMPO unit were further refined with DFT calculations at the level of ωB97xD/6-31G(d,p) theory using the Gaussian16 suite, version B.01.[7] The Packmol[8] and Moltemplate[9] packages were used for packing initial systems and generating LAMMPS input files. A real space cutoff distance of 10 Å was used for nonbonded interactions, which included truncated Lennard-Jones potential and Coulombic interactions. The long-range Coulombic interactions were treated by using the particle-particle particle-mesh solver with 10^{-4} accuracy. A Nose-Hoover barostat or thermostat was applied to control the pressure or temperature of the system. The timestep was set to 0.5 fs initially using a standard velocity-Verlet integrator during the pre-equilibration steps and then set to 1.0 fs after the system's temperature was increased (see below).

To initialize the simulation, 20 of TEMPO-containing 5-mers with different lengths of carbon spacing (n=1, 3, 4, 6, 9) and propylene carbonate molecules of the same mass with 5-mers were randomly placed within a cubic box with side lengths of 10 nm. At the beginning of the simulation, an energy minimization was performed to relax the system. Then, atoms in the box were initialized with a Gaussian-distributed velocity of 300 K. After the initialization, the system was kept at 300 K for 100 ps under the NVT ensemble, followed by 100 ps simulation under the NPT ensemble as pre-equilibration steps. Then, the system was heated up linearly to 1000 K for 0.5 ns, maintained at 1000 K for 0.5 ns, and cooled down linearly from 1000 K to 300 K for 2 ns to search for a lower energy configuration under the NVT ensemble throughout the process. The temperature was kept at 300 K and pressure at 1 bar under NPT ensemble for 5 ns as the production run. We repeated MD simulations for each 5-mer five times with different initialized velocities randomly.

Table S1. Peak and mean values of hopping distances (in units of Å) within 10 Å and the number of TEMPO dimers

Parameters	n=1	n=3	n=4	n=6	n=9
Hopping Dist. Peak	7.25	7.22	6.99	7.21	7.46
Hopping Dist. Mean	7.51	7.64	7.77	7.62	7.80
Counts	37126	25855	25714	18057	15519

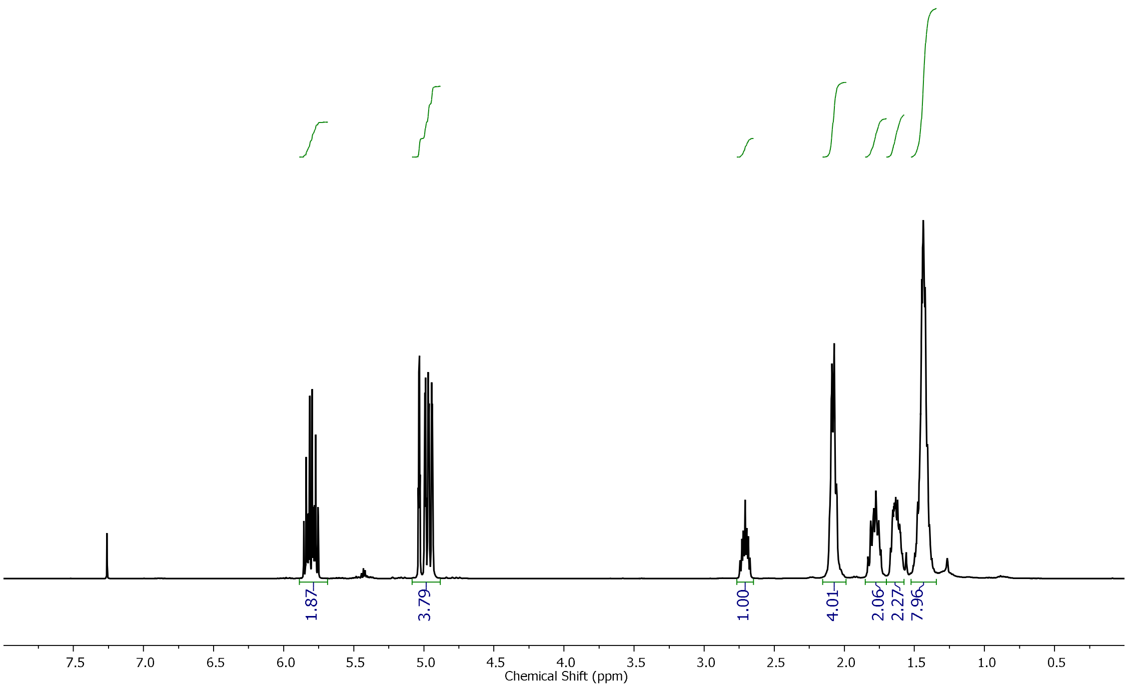

Figure S1. ^{1}H NMR spectroscopy of 1b

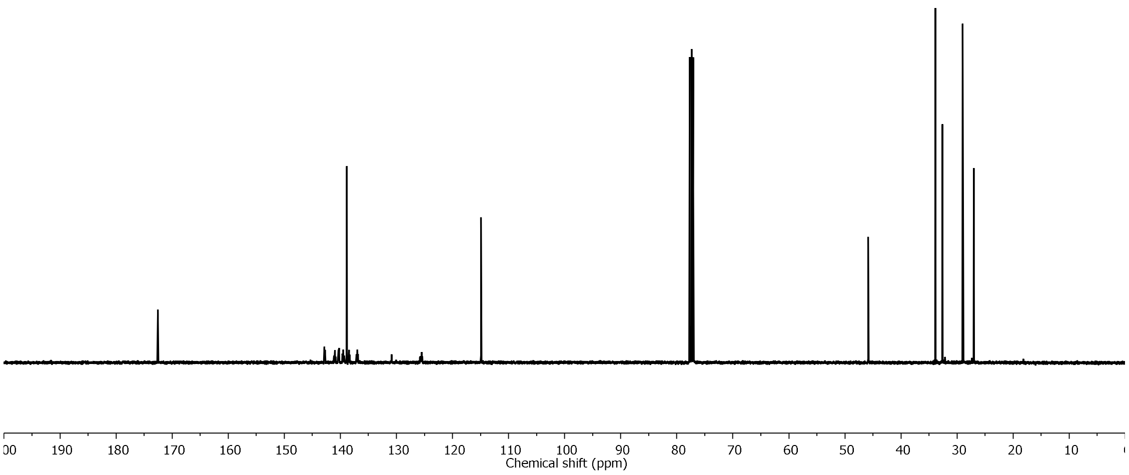

Figure S2. ^{13}C NMR spectroscopy of 1b

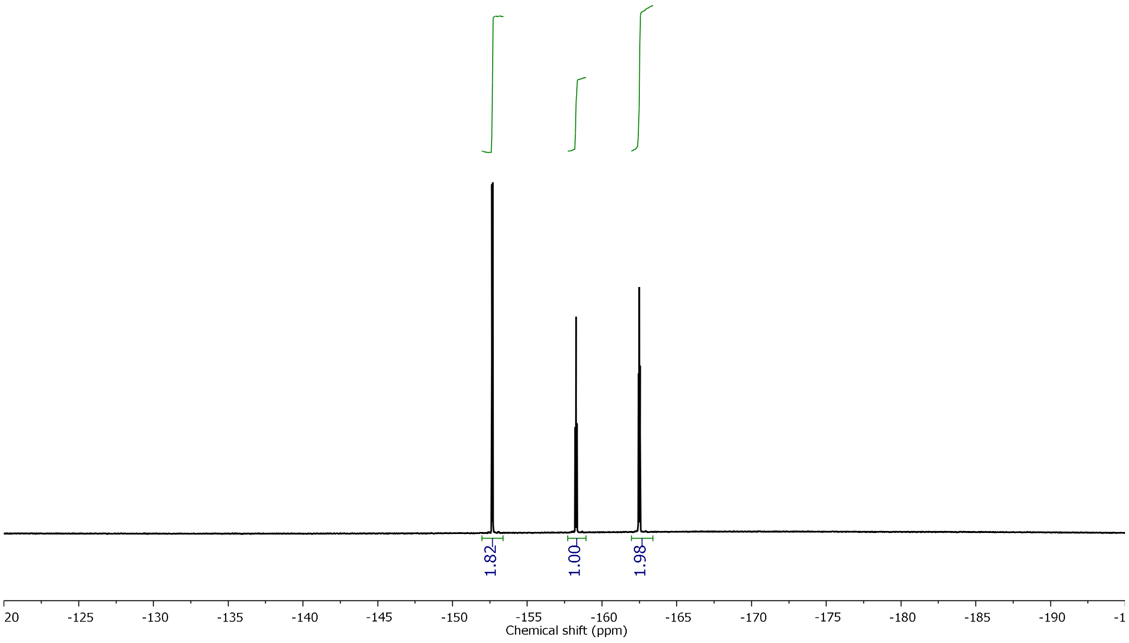

Figure S3. ^{19}F NMR spectroscopy of 1b

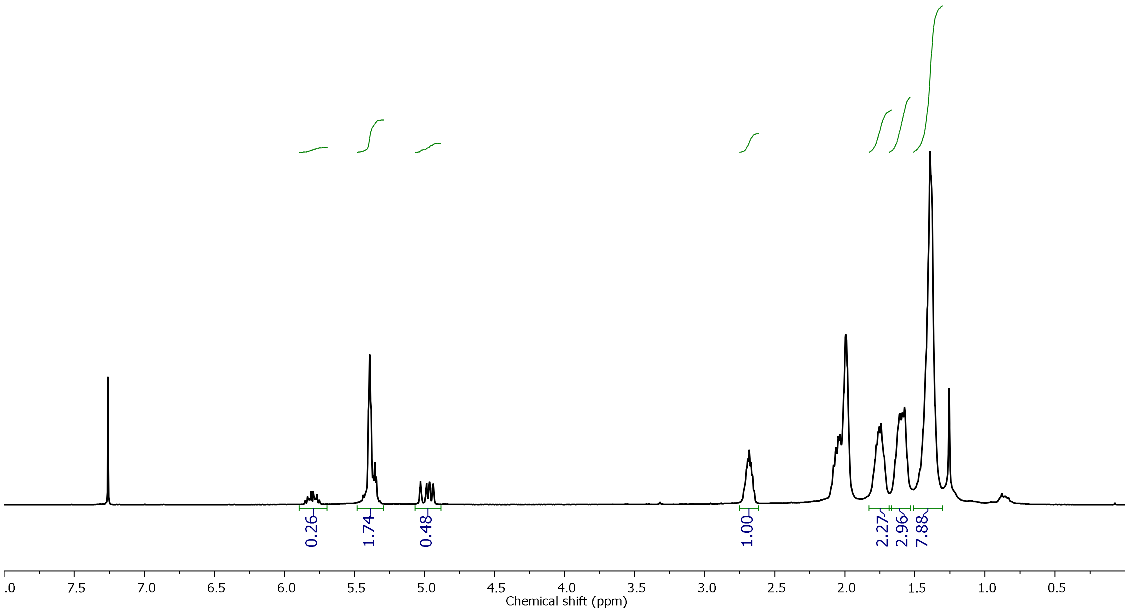

Figure S4. ^{1}H NMR spectroscopy of 2b

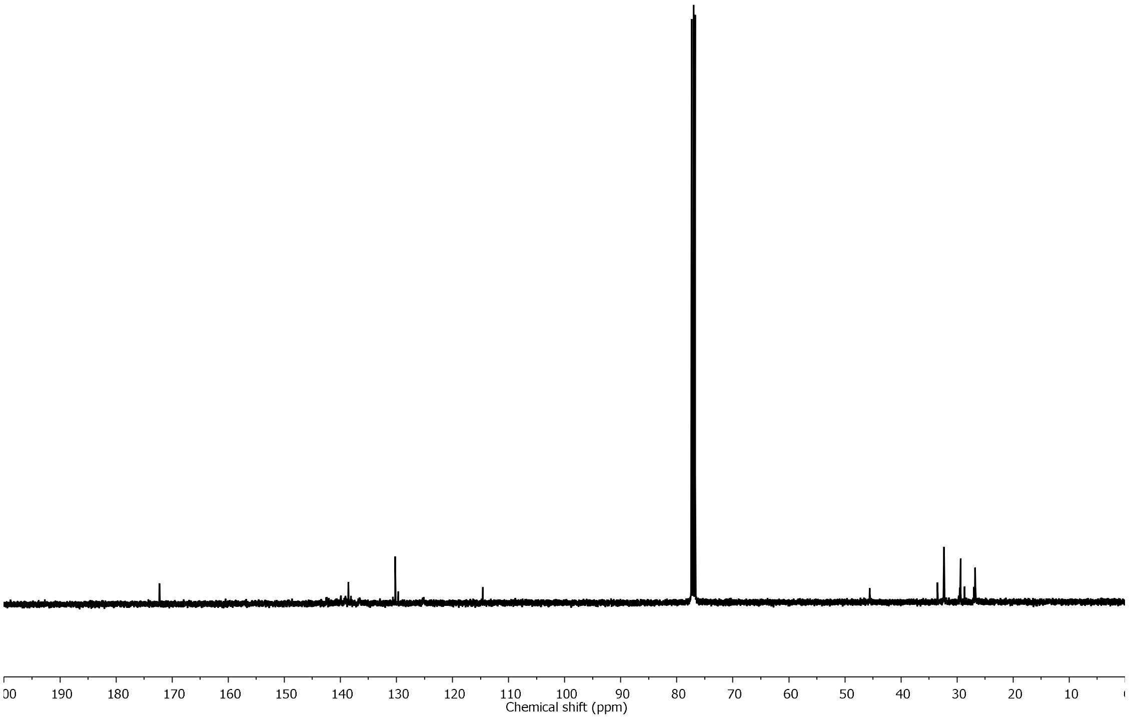

Figure S5. ^{13}C NMR spectroscopy of 2b

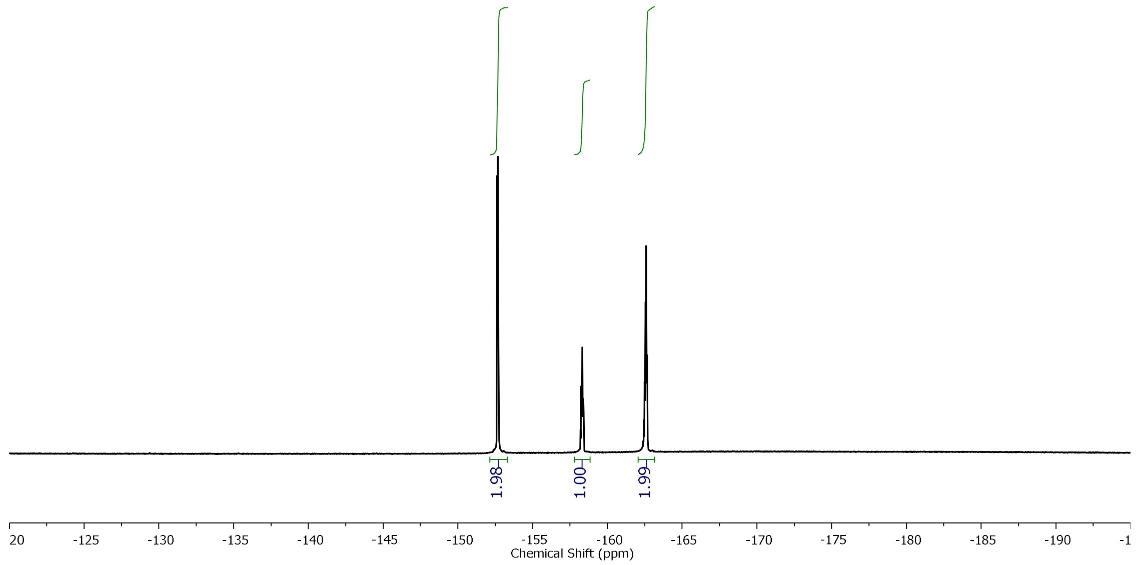

Figure S6. ^{19}F NMR spectroscopy of 2b

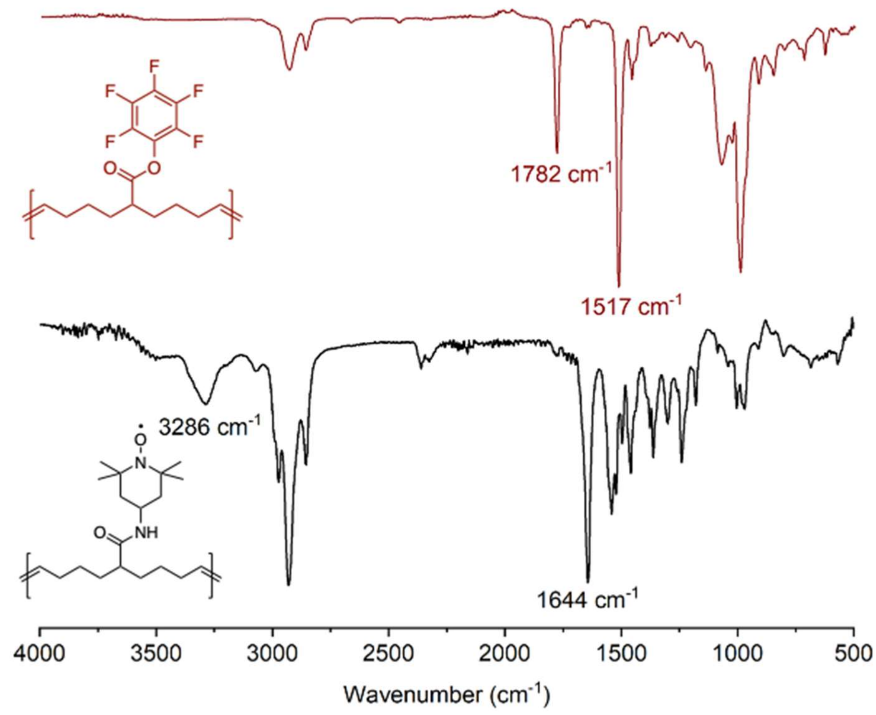

Figure S7. FTIR spectra of **2a** (top) and **3a** (bottom).

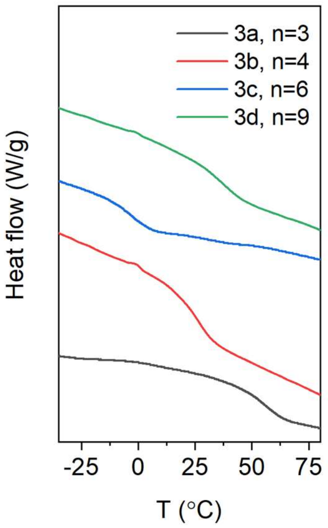

Figure S8. DSC thermograms of each polymer. All data are for the second heating ramp in a heat-cool-heat-cool cycle and were collected at a 10 °C/min ramp rate.

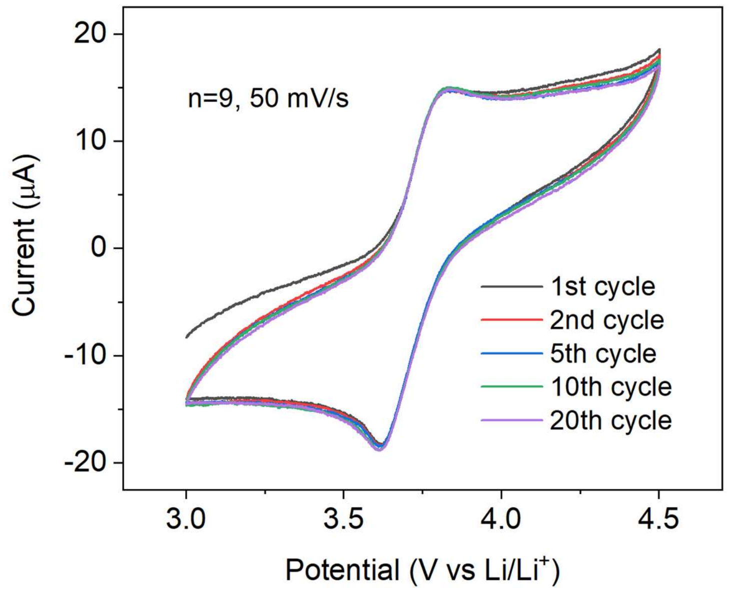

Figure S9. Cyclic voltammetry of the TEMPO-containing polymers with varying radical spacings of n=9. The electrolyte was 0.5 M LiOTf in propylene carbonate. Li metal strips were used as the counter and reference electrodes.

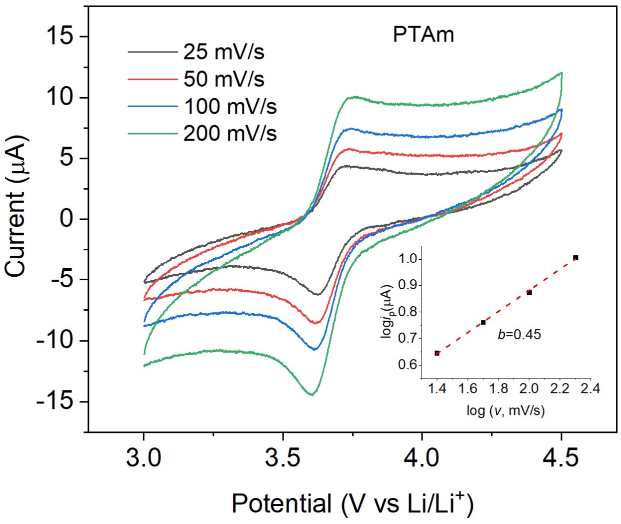

Figure S10. Cyclic voltammetry of PTAm. The electrolyte was 0.5 M LiOTf in propylene carbonate. Li metal strips were used as the counter and reference electrodes. Insets show the log-log plot of the peak current versus scan rate to obtain the *b*-value of PTAm.

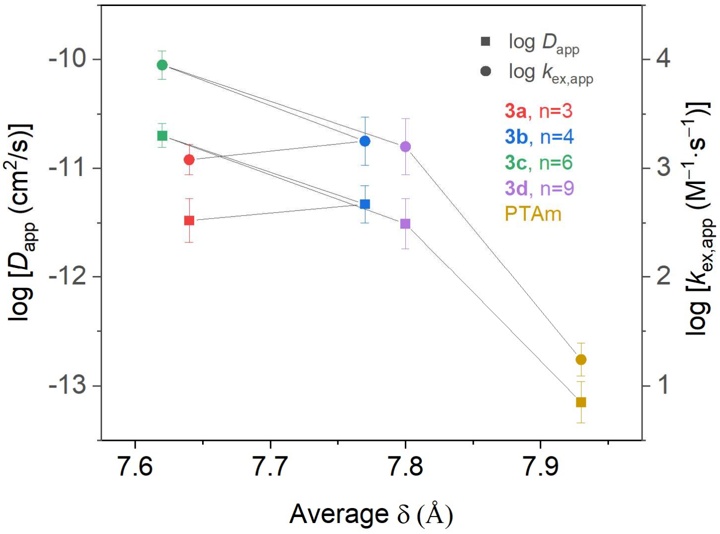

Figure S11. Comparison of log[D_{app}] and log[$k_{ex,app}$] with average hopping distance δ for polymers of different radical spacing. The data points are the mean value estimated from three measurements and the error bar is the standard deviation.

References:

1. Ma, T.; Easley, A. D.; Thakur, R. M.; Mohanty, K. T.; Wang, C.; Lutkenhaus, J. L., Nonconjugated Redox-Active Polymers: Electron Transfer Mechanisms, Energy Storage, and Chemical Versatility. *Annu Rev Chem Biomol Eng* **2023,** *14*, 187-216.
2. Zhang, K.; Xie, Y.; Noble, B. B.; Monteiro, M. J.; Lutkenhaus, J. L.; Oyaizu, K.; Jia, Z., Unravelling kinetic and mass transport effects on two-electron storage in radical polymer batteries. *Journal of Materials Chemistry A* **2021,** *9* (22), 13071-13079.
3. Sato, K.; Ichinoi, R.; Mizukami, R.; Serikawa, T.; Sasaki, Y.; Lutkenhaus, J.; Nishide, H.; Oyaizu, K., Diffusion-Cooperative Model for Charge Transport by Redox-Active Nonconjugated Polymers. *J Am Chem Soc* **2018,** *140* (3), 1049-1056.
4. Thompson, A. P.; Aktulga, H. M.; Berger, R.; Bolintineanu, D. S.; Brown, W. M.; Crozier, P. S.; in 't Veld, P. J.; Kohlmeyer, A.; Moore, S. G.; Nguyen, T. D.; Shan, R.; Stevens, M. J.; Tranchida, J.; Trott, C.; Plimpton, S. J., LAMMPS - a flexible simulation tool for particle-based materials modeling at the atomic, meso, and continuum scales. *Computer Physics Communications* **2022,** *271*.
5. Banks, J. L.; Beard, H. S.; Cao, Y.; Cho, A. E.; Damm, W.; Farid, R.; Felts, A. K.; Halgren, T. A.; Mainz, D. T.; Maple, J. R.; Murphy, R.; Philipp, D. M.; Repasky, M. P.; Zhang, L. Y.; Berne, B. J.; Friesner, R. A.; Gallicchio, E.; Levy, R. M., Integrated Modeling Program, Applied Chemical Theory (IMPACT). *J Comput Chem* **2005,** *26* (16), 1752-80.
6. *Schrödinger Release 2020-1, Schrödinger, LLC, New York, 2020.*
7. Frisch, M. J. T., G. W.; Schlegel, H. B.; Scuseria, G. E.; Robb, M. A.; Cheeseman, J. R.; Scalmani, G.; Barone, V.; Petersson, G. A.; Nakatsuji, H.; Li, X.; Caricato, M.; Marenich, A. V.; Bloino, J.; Janesko, B. G.; Gomperts, R.; Mennucci, B.; Hratchian, H. P.; Ortiz, J. V.; Izmaylov, A. F.; Sonnenberg, J. L.; Williams-Young, D.; Ding, F.; Lipparini, F.; Egidi, F.; Goings, J.; Peng, B.; Petrone, A.; Henderson, T.; Ranasinghe, D.; Zakrzewski, V. G.; Gao, J.; Rega, N.; Zheng, G.; Liang, W.; Hada, M.; Ehara, M.; Toyota, K.; Fukuda, R.; Hasegawa, J.; Ishida, M.; Nakajima, T.; Honda, Y.; Kitao, O.; Nakai, H.; Vreven, T.; Throssell, K.; Montgomery, J. A., Jr.; Peralta, J. E.; Ogliaro, F.; Bearpark, M. J.; Heyd, J. J.; Brothers, E. N.; Kudin, K. N.; Staroverov, V. N.; Keith, T. A.; Kobayashi, R.; Normand, J.; Raghavachari, K.; Rendell, A. P.; Burant, J. C.; Iyengar, S. S.; Tomasi, J.; Cossi, M.; Millam, J. M.; Klene, M.; Adamo, C.; Cammi, R.; Ochterski, J. W.; Martin, R. L.; Morokuma, K.; Farkas, O.; Foresman, J. B.; Fox, D. J. Gaussian16 Revision B.01, 2016.
8. Martinez, L.; Andrade, R.; Birgin, E. G.; Martinez, J. M., PACKMOL: a package for building initial configurations for molecular dynamics simulations. *J Comput Chem* **2009,** *30* (13), 2157-64.
9. Jewett, A. I.; Stelter, D.; Lambert, J.; Saladi, S. M.; Roscioni, O. M.; Ricci, M.; Autin, L.; Maritan, M.; Bashusqeh, S. M.; Keyes, T.; Dame, R. T.; Shea, J. E.; Jensen, G. J.; Goodsell, D. S., Moltemplate: A Tool for Coarse-Grained Modeling of Complex Biological Matter and Soft Condensed Matter Physics. *J Mol Biol* **2021,** *433* (11), 166841.
